# Identification and Characterization of a Novel Monoterpene Synthase from Soybean Restricted to Neryl Diphosphate Precursor

**DOI:** 10.1371/journal.pone.0075972

**Published:** 2013-10-04

**Authors:** Man Zhang, Jianyu Liu, Kai Li, Deyue Yu

**Affiliations:** 1 National Center for Soybean Improvement, National Key Laboratory of Crop Genetics and Germplasm Enhancement, Nanjing Agricultural University, Nanjing, Jiangsu, China; 2 Institute of Vegetable Crops, Jiangsu Academy of Agricultural Sciences, Nanjing, Jiangsu, China; Key Laboratory of Horticultural Plant Biology (MOE), China

## Abstract

Terpenes are important defensive compounds against herbivores and pathogens. Here, we report the identification of a new monoterpene synthase gene, *GmNES*, from soybean. The transcription of *GmNES* was up-regulated in soybean plants that were infested with cotton leafworm (*Prodenia litura*), mechanically wounded or treated with salicylic acid (SA). Gas chromatography-mass spectrometry (GC-MS) analysis revealed that recombinant GmNES enzyme exclusively produced nerol, generated from a newly identified substrate for monoterpene synthase: neryl diphosphate (NPP). This finding indicates that *GmNES* is a nerol synthase gene in soybean. Subcellular localization using GFP fusions showed that GmNES localized to the chloroplasts. Transgenic tobacco overexpressing *GmNES* was generated. In dual-choice assays, the *GmNES*-expressing tobacco lines significantly repelled cotton leafworm. In feeding tests with transgenic plants, the growth and development of cotton leafworm were significantly retarded. This study confirms the ecological role of terpenoids and provides new insights into their metabolic engineering in transgenic plants.

## Introduction

Terpenes are one of the most common groups of induced volatile plant compounds, with tens of thousands of structures and a broad variety of functions. In plants, the well-known role of terpenes is in the interaction between plants and the environment, with terpenes serving as defensive compounds against herbivores and pathogens [1]. The wide spectrum of terpenes’ indirect defense activities has been well investigated, i.e. terpenoid compounds can act as pollinator attractants [2], or feeding deterrents or insect toxins [3], [4], and are toxic to bacteria [5] and fungi [6].

Terpenoids are synthesized from the C_5_ building blocks isopentenyl diphosphate (IPP) and dimethyl allyl diphosphate (DMAPP). Two independent biosynthetic pathways can produce IPP: the mevalonate (MVA) pathway localized to the cytosol, and the 2C-methyl erythritol 4-phosphate (MEP) pathway localized to plastids [7]. Although these two pathways function independently, there is bounds of evidence that crosstalk occurs [8]. Based on the number of C_5_ units, terpenes are classified into hemiterpenes (C_5_), monoterpenes (C_10_), sesquiterpenes (C_15_), and diterpenes (C_20_) [9]. Geranyl diphosphate (GPP) is the widely accepted common substrate for monoterpene biosynthesis. Previous labeling studies showed that conversion of GPP to its *cis*-isomer neryl diphosphate (NPP) is not necessary before cyclization [10]. However, research on monoterpene in tomato glands contradicted the traditional view of GPP, proving that NPP could serve as a precursor for the synthesis of monoterpenes [11]. Similarly, farnesyl diphosphate (FPP) and geranylgeranyl diphosphate (GGPP) are two common substrates for sesquiterpene and diterpene biosynthesis, respectively. The corresponding substrates are converted into a wide range of terpenes by the action of terpene synthases [12].

To date, terpene synthases have been identified and characterized in many species including *Arabidopsis* [13], *Medicago truncatula* [14], [15], and *Lotus japonicus* [16]. However, little is known about the enzyme in soybean (*Glycine max* L. Merr.), one of the most important legume plants. In our previous work, we successfully isolated the *DXS* and *DXR* genes, which are the committed enzymes of the MEP pathway, from soybean [17], [18], indicating that the MEP pathway may play an important role in soybean self-defense. Until now, however, no monoterpene synthases have been characterized in soybean. In this work, we describe a novel gene, designated as *GmNES*, that encodes a nerol synthase that acts on NPP instead of the common substrate GPP for monoterpene biosynthesis. The expression and subcellular localization of *GmNES*, and the effects of *GmNES*-overexpressing plants on insects are also examined.

## Results

### Identification and Cloning of the *GmNES* Gene

First, known monoterpene synthases (mono-TPSs) were used to screen the soybean expressed sequence tag (EST) database to identify homologous sequences. A 1106 bp EST contig for a putative mono-TPS was identified. To determine the 5′ and 3′ ends, multiple rounds of 5′ rapid identification of cDNA ends (RACE) and 3′-RACE were performed, which resulted in 750 bp and 350 bp sequence fragments, respectively. Based on these two fragments and the previous partial sequence, PCR primers were then designed to amplify the full-length cDNA sequence, named *GmNES,* which was deposited in GenBank (accession number JF758895). *GmNES* encodes a predicted protein of 565 amino acids (aa), with a calculated molecular mass of 66 kDa and a predicted pI of 5.6. The cDNA sequence was further aligned with the soybean genome sequence (http://www.phytozome.net), and the organization of the *GmNES* gene was revealed, showing that the *GmNES* gene maps to chromosome 13 and contains six exons and five introns with a total length of 3.7 kb.

**Figure 1:**
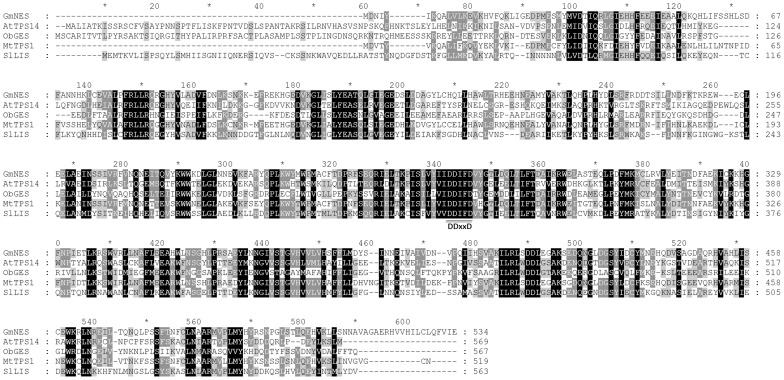
GmNES sequence alignment. The GmNES sequence was aligned with proteins from *Arabidopsis thaliana* (AtTPS14, NM_001198357), *Medicago truncatula* (MtTPS1, ABE80835), *Ocimum basilicum* (ObGES, AY362553) and *Solanum lycopersicum* (SlLIS, AEP82767). Residues shaded in black indicate conserved identical residues in the three sequences shown, and residues shaded in gray are identical in at least two of the three sequences shown. The DDxxD motif is indicated by a double horizontal line.

To characterize the sequence of *GmNES*, the protein sequence of GmNES was aligned with the sequences of certain known plant monoterpene synthases. As shown in Figure 1, GmNES contained a predicted N-terminal transit peptide-like sequence for chloroplast targeting [19] and a conserved DDxxD motif that is crucial for divalent cation (typically Mg^2+^ or Mn^2+^)-assisted substrate binding [20]. However, GmNES lacked the RRx_8_W motif, which is characteristic of most monoterpene synthase members of the subfamily Tps-b [21] and is proposed to be involved in cyclizing monoterpene synthases. Based on recent research, the conserved RRx_8_W motif is thought to be required for the use of GPP as a substrate [22]. However, this motif may not be required for the formation of acyclic monoterpenes.

**Figure 2:**
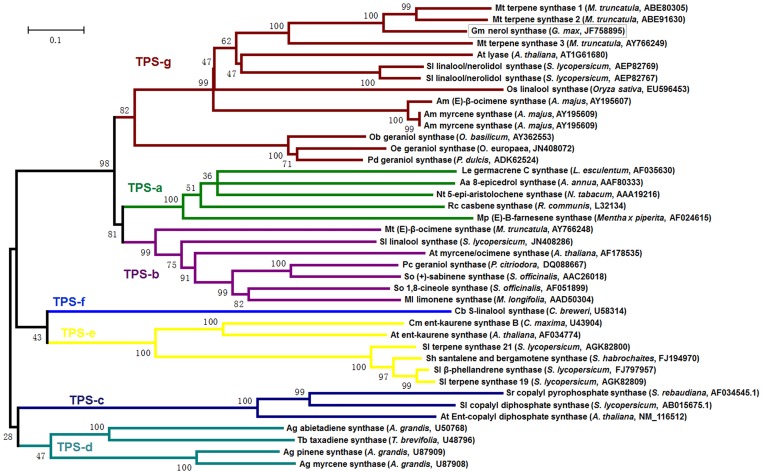
Phylogenetic analysis of GmNES (shown in the box) and other plant terpene synthases. Sequence analysis was performed using Clustal X2.1. MEGA 4 was applied to create trees using the nearest neighbor-joining method (1,000 replicates for bootstrap values). The plastid targeting signal peptides were not excluded from the analysis. The scale bar indicates a 10% change in amino acids. GenBank accession numbers are also given for each peptide sequence.

A phylogenetic tree was constructed to determine the evolutionary relationship of GmNES with other known terpene synthases from both plants and microorganisms. The result showed that GmNES belongs to the TPS-g subfamily (Figure 2) [9], [21]. Southern blotting analysis was conducted to detect the copy number of *GmNES* in the soybean genome and a single band was obtained (data not shown), suggesting that *GmNES* exists as a single-copy gene.

### Expression Profile Analysis of *GmNES*

**Figure 3:**
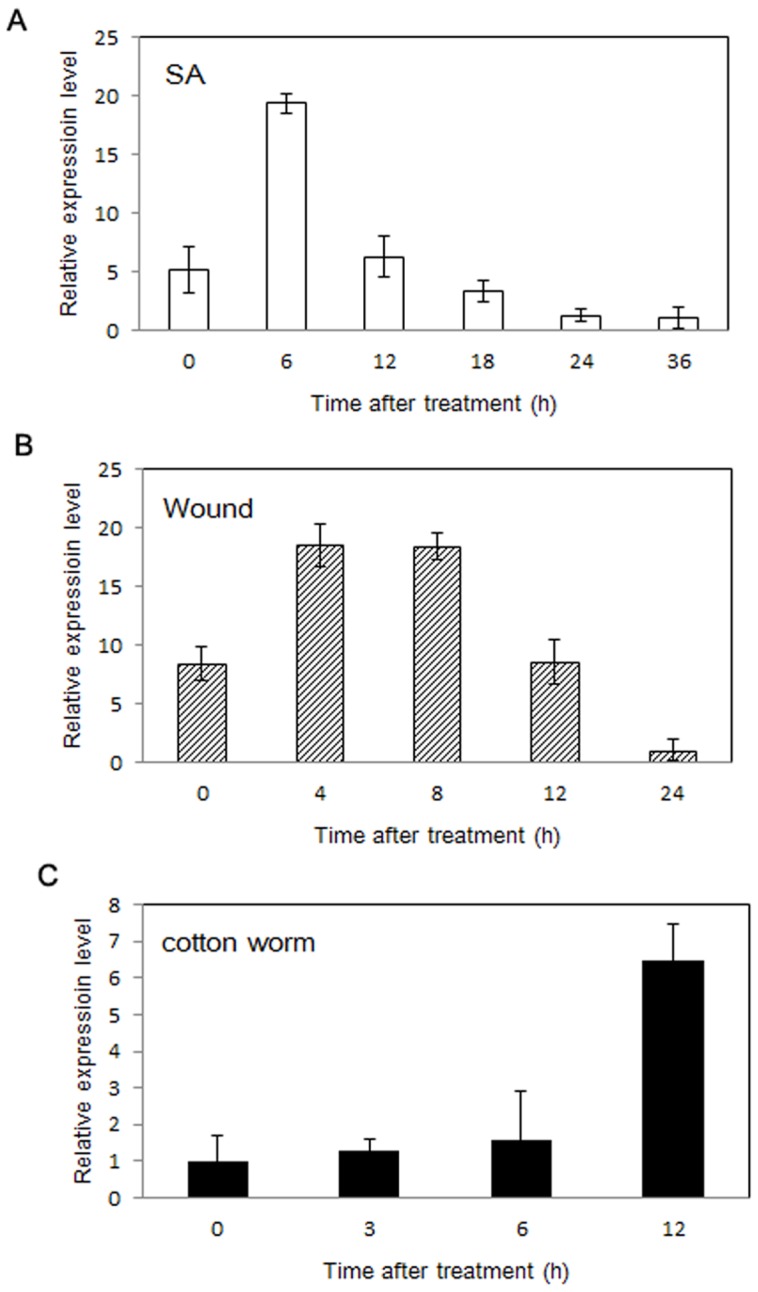
Real-time quantitative PCR analysis of *GmNES* transcription. Total RNA was extracted from the leaves of soybean under constitutive conditions (control), and after treatment with an aqueous solution of salicylic acid (**a**), after mechanical wounding (**b**) and after feeding by cotton leafworm larvae (**c**). The soybean *actin* gene was used as control.

To gain insight into the expression profile of *GmNES*, we tested the expression of *GmNES* in soybean leaves under different conditions, such as treatment with plant signaling molecules, mechanical wounding and feeding by cotton leafworm larvae. Transcripts of *GmNES* greatly accumulated at 6 h after treatment with salicylic acid (SA) and then gradually decreased until the end of the experiment (Figure 3A). Transcripts of *GmNES* were induced at 12 h after cotton leafworm treatment (Figure 3C). However, the expression profile induced by mechanical wounding was different, resulting in an induction of *GmNES* transcription 4 h after wounding, which reached a peak of expression at 8 h, followed by a reduction (Figure 3B). These results suggest that herbivore feeding, mechanical wounding and the application of exogenous SA stimulate the up-regulation of *GmNES* expression, although with different transcript levels [16].

### Functional Characterization of GmNES

**Figure 4:**
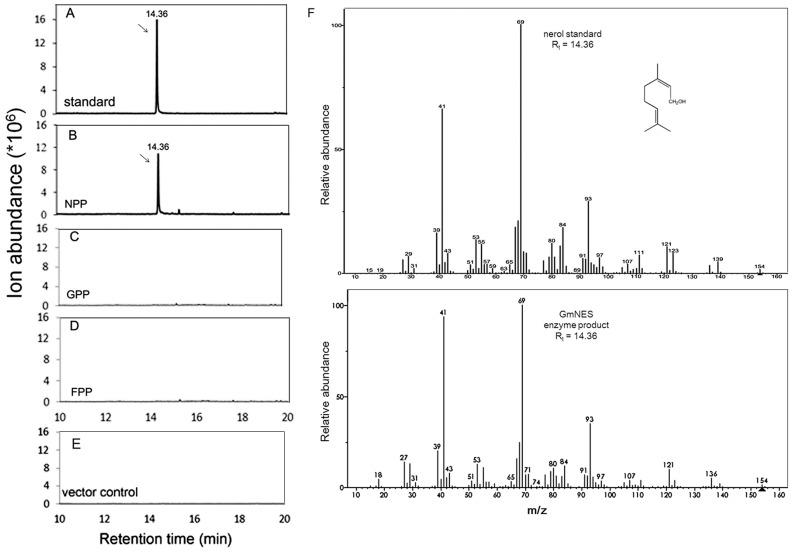
*In vitro* assay of recombinant GmNES with different substrates by GC-MS analysis. The expression of truncated GmNES cDNA in *E. coli* resulted in the synthesis of a monoterpene product formed from NPP. (**a**) Authentic nerol standard. (**b**) Purified recombinant GmNES incubated with NPP. (**c**) Purified recombinant GmNES incubated with GPP. (**d**) Purified recombinant GmNES incubated with FPP. (**e**) Vector incubated with NPP as control. (**f**) Mass spectra of the enzyme product and the reference substance nerol. The insert shows the structure of the product nerol.

For the functional characterization of GmNES, a truncated cDNA fragment was subcloned into the pDEST-17 expression vector and then expressed in the *E. coli* strain BL21-AI. The affinity-purified protein was assayed using three different prenyl diphosphate substrates: GPP, FPP and NPP. The products were analyzed by gas chromatography-mass spectrometry (GC-MS). As shown in Figure 4B, only assays with NPP as the substrate exclusively yielded a monoterpene hydrocarbon product, which was identified as nerol using authentic standards for the comparison of retention times (Figures 4A, 4B) and mass spectra (Figure 4F). In contrast, a control, which was prepared from *E. coli* BL21-AI harboring pDEST-17 without the *GmNES* insert, did not produce any monoterpene products (Figure 4E). While GmNES recombinant enzyme was inactive when GPP or FPP was used as substrate (Figures 4C, 4D), neither the vector control (Figure 4E). Overall, these data indicate that GmNES is a monoterpene synthase that exclusively produces nerol in the presence of NPP.

### Subcellular Localization of GmNES

**Figure 5:**
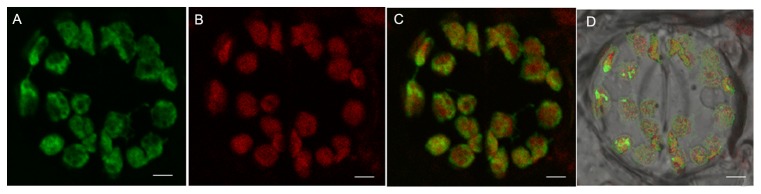
Subcellular localization of GmNES. GmNES was fused to the N-terminus of GFP and the fusion protein was transformed into tobacco. Transgenic plants expressing the fusion protein were analyzed by laser confocal microscopy. (**a**) Green fluorescence of GmNES::GFP. (**b**) Red autofluorescence of chlorophyll. (**c**) Merged images of (**a**) and (**b**). (**d**) Bright field images. Bars = 10 µm.

Monoterpene synthesis is believed to primarily occur in plastids. GC-MS analysis revealed that GmNES acts as a monoterpene synthase. The presence of an N-terminal cTP predicted that GmNES is located in the chloroplast. To confirm the subcellular localization of GmNES, the *GmNES* full-length cDNA was fused to *GFP* and then transferred into tobacco by *Agrobacterium*-mediated transformation. GFP expression was analyzed by confocal laser scanning microscopy (Figure 5). The GFP fluorescence of the GmNES::GFP fusion protein was observed exclusively in the chloroplast (Figure 5A). The result confirmed the predicted plastid localization of GmNES.

### Transgenic Tobacco Plants Expressing the *GmNES* Gene Produce Nerol

**Figure 6:**
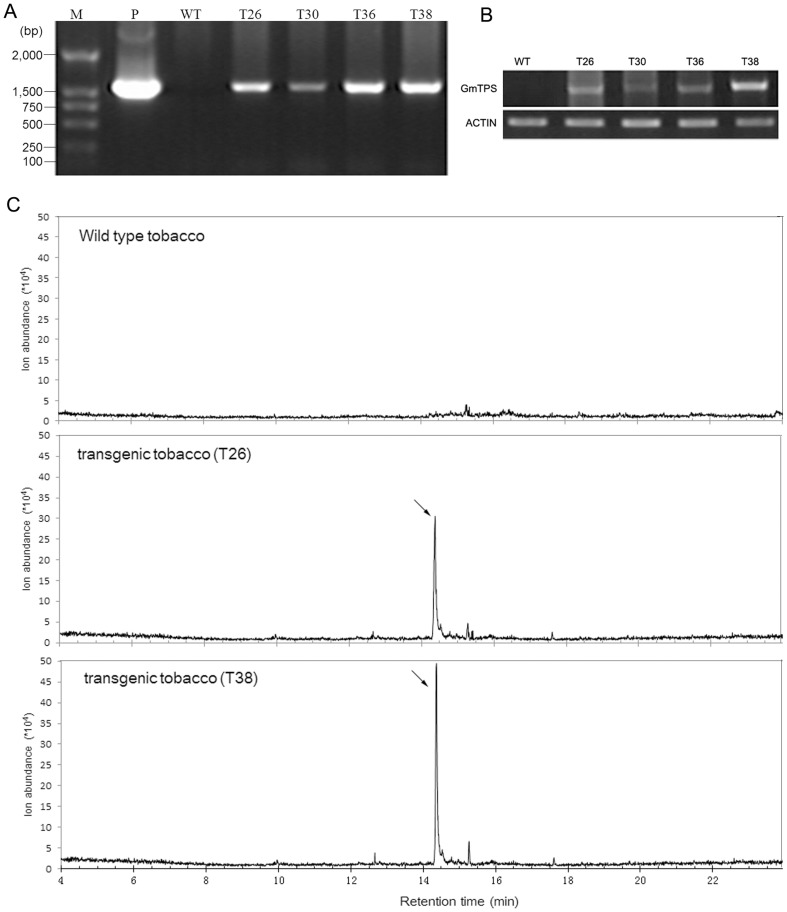
Headspace measurement of leaves of transgenic tobacco plants overexpressing *GmNES* gene. (**a**) PCR assay of the *GmNES* gene in transgenic tobacco. M: DL 2,000 DNA Marker; P: positive control; WT: wild-type tobacco; T26–T38: transgenic tobacco over-expressing *GmNES* gene. (**b**) RT-PCR assay of *GmNES* gene in transgenic tobacco. (**c**) Varying amounts of nerol were detected in the headspace of the transgenic plant overexpressing *GmNES* (T26 and T38 for example) but not in the wild-type control.

To demonstrate the potential of tobacco for the heterologous expression of terpenes, a construct containing the *GmNES* open reading frame under the control of the 35S promoter of *Cauliflower mosaic virus* (CaMV) was used for the transformation of tobacco. Transgenic plants were generated via the *Agrobacterium*-mediated transformation method. Resistance to hygromycin was used for selecting putative transgenic plants. The hygromycin-resistant plants were further examined by PCR for the presence of the *GmNES* gene (Figure 6A) and by RT-PCR for the transcription of the *GmNES* gene (Figure 6B). Two leaves from each individual transgenic plant were screened for terpenoid emission. As expected, the leaves of wild-type tobacco did not produce any detectable nerol (Figure 6C, wild-type tobacco); whereas, the transgenic lines showed varying levels of nerol emission (Figure 6C, transgenic tobacco).

### The Behavior of Cotton Leafworm is Influenced by Transgenic Plants Expressing GmNES

**Figure 7:**
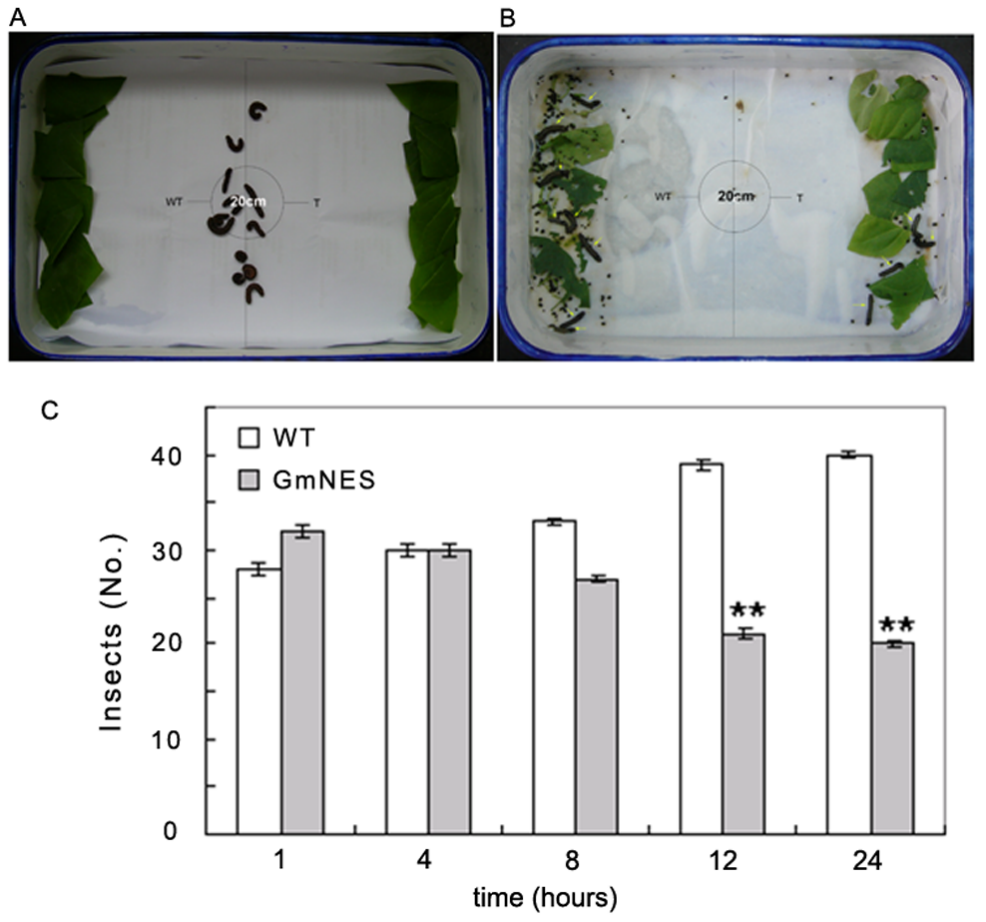
Behavior of cotton leafworm larvae is influenced by transgenic plants overexpressing *GmNES*. (**a**) Dual-choice assay of cotton leafworm. Twelve insects were used for each replication, with five replicates in total. (**b**) Twelve hours after incubation, eight insects were in side of the wild-type plants (WT) and four were in side of the transgenic tobacco plants expressing *GmNES* (T). (**c**) The preference of cotton leafworm for detached WT and T plants. **means a significant difference at the level of *p*<0.01.

Terpenes play an important role in plant defense by either attracting or repelling herbivores. In this study, GmNES exclusively used NPP as substrate to produce the monoterpene nerol, so, we overexpressed the *GmNES* gene in tobacco to estimate the gene’s effect on the behavior of cotton leafworm (an important soybean pest in southern China). The preference of cotton leafworm for the detached leaves of transgenic and wild-type plants was investigated in a dual-choice assay (Figures 7A, 7B). The results showed that over time, the cotton leafworm larvae significantly preferred the wild-type tobacco leaves to the *GmNES*-expressing tobacco leaves (Figure 7B).

### The Growth of Cotton Leafworm is Significantly Inhibited by Feeding on Transgenic Tobacco Plants Expressing GmNES

**Figure 8:**
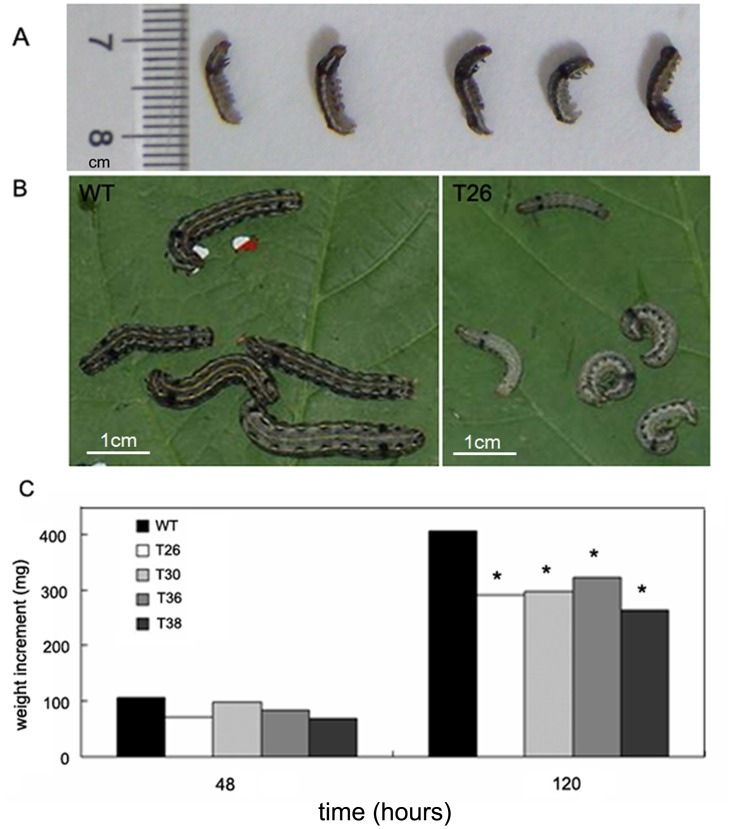
Growth inhibition of cotton leafworm by feeding on transgenic plants expressing *GmNES* (T) compared with wild-type plants (WT). (**a**) Third-instar cotton leafworm larvae. Five larvae were used for each repeat, with a total of four replicates for each plant examined. (**b**) Images at 120 h after feeding. Left: larvae feeding on the leaves of wild-type plants. Right: larvae feeding on the transgenic plant overexpressing *GmNES* (T26 as an example). (**c**) Weight increase at 48 and 120 h after feeding. The data represent the mean values of five replicates ± standard deviation. Significant differences from controls are indicated by * at the level of *p*<0.05.

In addition to the dual-choice assay, a feeding test experiment was designed to further determine the influence of the monoterpene nerol on insects’ growth and development. The weight gain of cotton leafworm larvae feeding on the leaves of transgenic plants expressing *GmNES* or wild-type plants served as the index for evaluation. The results showed that larvae feeding on transgenic tobacco plants grew less vigorously than their nontransgenic counterparts over time (Figure 8C). The growth of cotton leafworm larvae was retarded, leading to lower weight gain (Figure 8B). There was no significant differences between the four transgenic lines analyzed (Figure 8C).

## Discussion

Here, we report a monoterpene synthase gene, *GmNES*, that can convert neryl diphosphate (NPP) precursor rather than geranyl diphosphate (GPP) into the new monoterpene product nerol in soybean (Figure 3). Several aspects of the *GmNES* gene have been addressed in the present work: the enzymatic activity of the recombinant protein, the expression profile of the gene, the subcellular localization of the encoded protein and the ecological significance of *GmNES*-expressing plants.

Both geranyl diphosphate (GPP) and neryl diphosphate (NPP) can be used as substrates for monoterpene synthases. However, it is widely accepted that GPP is the ‘universal’ substrate of monoterpene synthases [23] because these enzymes can perform the necessary isomerization before cyclization [24], and several studies have described the use of GPP by monoterpene synthases [15], [16]. Using a cell-free assay it was demonstrated that NPP can be converted into several monoterpenes [25]. However, no specific enzymes have been identified and no additional information on NPP was documented until Schilmiller et al. [11] reported their findings. The authors discovered the *NDPS1* gene, encoding an NPP synthase, in tomato, and the *PHS1* gene, encoding phellandrene synthase, which uses NPP as substrate to produce monoterpenes, was also reported. These results provided evidence that NPP is indeed a substrate for monoterpene synthases. In agreement with these findings, we demonstrated that NPP can be used by the monoterpene synthase GmNES to catalyze the formation of nerol (Figure 4B), further supporting NPP’s role as a substrate. The difference between our results and those of Schilmiller et al. [11] is that the major product of GmNES with NPP, nerol, is acyclic which is the same as the products of GPP, whereas the major products of PHS1 with NPP are cyclic. Hence, we could not conclude that the products of NPP are cyclic and/or that those of GPP are acyclic. To explain this discrepancy, the reaction mechanism of terpene synthases needs to be further investigated. Additionally, because the *NDPS1* gene, encoding an NPP synthase, was the first reported enzyme of this type [11], [23], to identify a potential NPP synthase in soybean, we screened the fully sequenced database of the *Glycine max* genome using *NDPS1* as the query sequence, which resulted in several sequences with high similarity (>80%). The BLAST result provides further evidence that indicates the existence of NPP in soybean. Subsequent gene cloning, protein expression and activity assays for soybean NPP synthases will be performed in the future.

Previous phylogenetic analyses of plant TPS protein sequences revealed seven TPS gene subfamilies, designated Tps-a through Tps-g [21], [26], [27]. The identified soybean nerol synthase GmNES is closely related to *Medicago* terpene synthases and *Arabidopsis* AtTPS14 (At1g61680). These synthases, together with the snapdragon monoterpene synthases, were previously defined as the TPS-g subfamily, indicating that GmNES is a member of the TPS-g family (Figure 2). Lacking the RRx_8_W motif and the prevalence of acyclic products are the two prominent features of the members of the TPS-g group [26], [27]. According to sequence alignment and functional characterization, the RRx_8_W motif was missing in GmNES (Figure 1), and GmNES specifically produced acyclic monoterpene nerol from the substrate NPP (Figure 4). Interestingly, PHS [11], TPS19 from cultivated tomato (*Solanum lycopersicum*) [28] and *cis*-sesquiterpene TPS from wild tomato (*Solanum habrochaites*) (ShSBS) [29], which were previously shown to catalyze the formation of phellandrenes and several other monoterpenes and sesquiterpenes from NPP, are known to fall into the TPS-e subfamily. The phylogenetic result indicated that although the old substrates NPP could be used for catalyzing the formation of new enzymes for terpenoid biosynthesis, and although most of these terpene syntheses fell into the same subfamily (TPS-e subfamily), the substrate NPP could not be defined as a common feature of the enzymes in a TPS subfamily.

Multiproduction is an important feature of monoterpene synthases in many other plant species. For example, (*E*)-β-ocimene synthase in *M. truncatula* converts GPP into three monoterpenes, with (*E*)-β-ocimene as the major product, and trace amounts of two other compounds: (*Z*)-β-ocimene and myrcene [15]. PHS1 in tomato catalyzes NPP to form five monoterpenes, with β-phellandrene as the main product [11]. In contrast, GmNES can exclusively synthesize the monoterpene nerol from NPP (Figure 3). Among other characterized monoterpene synthases in plants, nerol synthase in soybean (GmNES) is the only identified enzyme that can produce nerol, which makes the enzyme atypical. Apart from multiproduction, another feature of terpene synthases is that different substrates (GPP or FPP) can be converted into corresponding terpene compounds by the same enzyme. AdAFS1, a sesquiterpene synthase identified in *Actinidia deliciosa*, can function as both a sesquiterpene synthase and a monoterpene synthase because AdAFS1 exclusively produces the sesquiterpene α-farnesene from FPP and the monoterpene (*E*)-β-ocimene from GPP [30]. In contrast, our results showed that *GmNES* could exclusively convert NPP and could not catalyze the formation of any monoterpenes and/or sesquiterpenes when GPP or FPP was employed (Figures 4C, 4D). Thus the specificity of the substrate and terpene product of *GmNES* should be of prime interest when strategies for the metabolic engineering of monoterpene biosynthesis in plants are considered for industrial utilization.

Monoterpenes and sesquiterpenes have been shown to be of ecological significance in plant defense [31], [32]. In this study, via dual-choice assay, we found that the monoterpene nerol, which is produced by *GmNES*-expressing plants, influences the behavior of cotton leafworm. Similar results were previously reported, showing the repellence of *M. persicae* aphids by linalool [3], [33]. In addition, based on feeding tests, we found that the growth and/or development of cotton leafworm was retarded when feeding on *GmNES*-expressing tobacco leaves compared with leafworm feeding on wild-type plants. These results suggested biological roles for the monoterpene nerol. One major explanation for the insects’ repellence and growth inhibition might be a toxic effect of the high level of nerol produced, as many monoterpenes have been demonstrated to be of ecological significance in plant defense [3], [31]. Our observation that nerol might repel cotton leafworm larvae from feeding on *GmNES*-expressing plants will be valuable in understanding direct plant defense against insect herbivores and useful for studying the indirect defense of plants, such as the altered behavior of insects caused by the production of specific volatile substances. Moreover, the hypothesis regarding the antimicrobial activity of terpenes [11] produced by enzymes of the MEP pathway and the effects of nerol on insect behavior indicate that the monoterpene nerol, synthesized by GmNES, could be a good source of plant insecticide.

Taken together, the results of the present study provide additional evidence that NPP is indeed a stable native monoterpene intermediate, revealing a way to discover new functions of terpene synthases and new products with substrate acceptance. This work will aid understanding of the diversity of the metabolic regulation and formation of terpene.

## Materials and Methods

### Plant Material, Insect and Microbial Maintenance

Soybean (*Glycine max* cv. ‘*Bogao’*) seeds were grown in the experimental field of Nanjing Agricultural University, Nanjing, P. R. China. Soybean organs (leaves, roots and stems) were excised when the first trifoliate leaf was well expanded. Flowers were collected at the stage of flowering (R_1_ stage). The pods (including the pod walls and seeds) were collected at 30 days after flowering. All tissues were separately immersed in liquid nitrogen and stored at −70°C untill use.

Cotton leafworm larvae were bought from Jiangsu Agricultural Research Institute, and maintained at room temperature on artificial media consisting of a wheat germ base.

### Cloning of *GmNES* Full-length cDNA by RACE

An EST sequence (TC211089) was identified after a BLAST search of the gene indices of The Institute of Genome Research (TIGR) (http://compbio.dfci.harvard.edu/tgi/), using known monoterpene synthases as the query. Based on the sequence information, the gene-specific primer pair *nes-f1* (5′-GCTATGTATGTTGCTAAGACTCTTCAGC-3′) and *nes-r1* (5′-AATATGCACTACATGTGCTCAGCAC-3′) was designed for PCR amplification using total RNA from *G. max* leaves as the template. The PCR program was as follows: 3 min at 94°C followed by 30 cycles of 30 s at 94°C, 50 s at 58°C, and 60 s at 72°C, then 10 min at 72°C. The PCR products were separated by electrophoresis in a 1.0% (w/v) agarose gel and visualized using a JS-380A automatic gel imaging analyzer (PeiQing, Shanghai, P. R. China). The amplified fragments were then subcloned into the pGEM-T Easy Vector (Promega, Madison, WI, USA) for sequencing. An internal 1106 bp DNA fragment amplified by the *nes-f1* and *nes-r1* primers, which showed homology to plant monoterpene synthases, was obtained.

RACE was performed with a SMART™ RACE cDNA Amplification Kit (Clontech, USA) to clone the 3′- and 5′-ends of *GmNES* cDNA following the manufacturer’s instructions. The 3′-end region was amplified by two nested PCR reactions. In the first-round PCR, the primers *nes3-f1* (5′-GCTTGTCCATTCATTCTTCCTC-3′) and *UPM* (supplied in the kit) were used with 3′-RACE-ready cDNA as a template. In the second-round PCR, the primers *nes3-f2* (5′-GGACTTGATGGGTCATACATTGA-3′) and *NUP* (supplied in the kit) were used with the first round products as a template. For 5′-RACE amplification, the two nested primers used were *nes5-r1* (5′-TCAGCAAGTTCCTCTAAGCATTC-3′) and *nes5-r2* (5′-GAAGAAGTTGGTGGCAGAGG-3′). Based on the sequence information obtained by the 5′- and 3′-RACE reactions, together with the internal sequence, the full-length cDNA was amplified by RT-PCR using the primers *nes-f1* (5′-GATGAGGCCAAAAATTGTGC-3′) and *nes-r1* (5′-GTGACATCTTTAAGTGCGTGGAC-3′) and then sequenced. The obtained full-length cDNA was designated *GmNES*.

The deduced amino acid sequence was aligned using the Clustal X2.1 program and edited using GeneDOC software (ver 2.6). The phylogenetic tree was created by using the neighbor-joining method and MEGA 4 software. Plastid-targeting peptides and cleavage sites were predicted by the ChloroP tool (ver 1.1).

### Plant Treatment for *GmNES* Gene Expression Analysis

‘*Bogao*’ seeds were grown in plastic pots (diameter = 8 cm, depth = 6.5 cm) containing five plants, which were kept in a growth chamber (12 h light/12 h dark, 25±1°C) for 2 months. To induce thermal stress, the temperature was maintained at constant level of 42°C. Leaf samples were then harvested at different time points (1, 3, 6, 9 and 12 h) after treatment and frozen in liquid nitrogen. To induce herbivorous insect infestation, five third-instar larvae of cotton leafworm, which had been starved for 4 h prior to the start of the experiments, were enclosed with the soybean plants in each pot and removed after 3 h. Leaf samples were collected 6, 12 and 24 h after treatment and frozen in liquid nitrogen. To test the effects of plant hormones, plants were sprayed with 1 mM SA (Invitrogen, USA) or water as a control. Leaf samples were collected 6, 12, 18, 24 and 36 h after treatment and frozen in liquid nitrogen.

### Gene Expression Analysis by Real-time Quantitative PCR

Total RNA was extracted using the RNApure Plant Kit with DNase I (CWBiotech, Beijing, China). First-strand cDNA was generated from 1.0 µg total RNA with an oligo (dT) primer from the BU-SuperScript RT Kit (Biouniquer, Beijing, P. R. China) according to the manufacturer’s instructions. The cDNA was used as a template for gene expression analysis. Real-time PCR was performed using the 1×SYBR Green PCR Master Mix (PE-Applied Biosystems, USA) and a GeneAmp® 7300 Sequence Detection System (PE-Applied Biosystems, USA) according to the manufacturer’s instructions. A 500-bp *GmNES* sequence fragment was amplified using *Pfu* DNA Polymerase (Promega, USA) with the primer pair *sd1* (5′-AGCATCCGCTTCATTATGACTT-3′) and *sd2* (5′-TAGGGTTGAATCCATGCTTCTT-3′). Soybean’s constitutively expressed *actin* gene (GenBank accession number V00450) was amplified as a control. The actin-specific primers were actin-F (5′-GAGAAATTGTCCGTGACATGAA-3′) and actin-R (5′-ATGGGCCAGACTCATCATATTC-3′) and the expected product was 486 bp in size. Three technical replicates were performed for each biological replicate. The relative gene expression levels were calculated using the 2^−ΔΔCT^ method [34]. SAS 9.2 software (SAS Institute, Cary, NC) was used for all statistical analyses [3].

### Expression of GmNES in *E*. *coli* and Enzyme Assays

A truncated *GmNES* cDNA fragment without the N-terminal transit plastid was cloned in the pDEST-17 vector (Invitrogen, Carlsbad, CA), and the construct was transformed into the *E. coli* strain BL21-AI. Incubation was performed at 18°C overnight with slight shaking. The cells were harvested by centrifugation, and the deposits were resuspended in extraction buffer (50 mM MOPS, pH 7.0, with 5 mM MgCl_2_, 5 mM sodium ascorbate, 0.5 mM PMSF, 5 mM dithiothreitol and 10% (v/v) glycerol) and disrupted with a Branson Sonifier 250 sonicator (Branson Ultrasonic Corporation, Danbury CT, USA) at inconstant power (approximately 5 W) for 30 s. The lysates were cleared by centrifugation, and the supernatants containing the soluble enzyme were purified by affinity chromatography using Ni-NTA resin. An enzyme assay was performed in a 1 ml volume containing 200 µl affinity-purified protein and 750 µl assay buffer (50 mM MOPS, pH 7.0, with 1 mM dithiothreitol and 10% (v/v) glycerol) with 10 µM geranyl diphosphate or farnesyl diphosphate (Echelon Research Laboratories, Salt Lake City, UT) or 10 µM neryl diphosphate (kindly provided by Dr. Charles Waechter (University of Kentucky, Lexington) and Dr. Jeffrey Rush (University of Kentucky, Lexington).

### Monoterpene Product Analysis by GC-MS

After incubation at 30°C for 60 min, a solid-phase microextraction (SPME) fiber consisting of 100 µm polydimethylsiloxane (SUPELCO, Belafonte, PA, USA) was placed into the headspace of a vial [35], which was then incubated at 30°C for 30 min. After incubation, the SPME fiber was directly inserted into the injector of a Thermo Finnigan (TRACE GC) gas chromatograph coupled to a Thermo Finnigan (TRACE DSQ) mass detector. Separation was performed on a DM-5 column (30 m × 0.25 mm i.d. × 0.25 µm thickness, DIKMA, China). A splitless injector was used at 200°C and a column flow of 1.0 ml He min^−1^. The temperature program was used as following: initial temperature was 40°C (3 min hold), and was then increased to 230°C (2 min hold) by a 10°C min^−1^ ramp. Products were identified by a comparison of retention time and mass spectra with those of authentic reference compounds, which were obtained from Sigma (Sigma-Aldrich, http://www.sigmaaldrich.com/).

### Transformation of Tobacco with 35s::GmNES

The Gateway system was used for *GmNES* overexpression vector construction. The entire coding sequence of the *GmNES* gene was amplified using primers that generated a gene-specific fragment with an *attB* site (*sense*: 5′-GGGGACAAGTTTGTACAAAAAAGCAGGCTTCGACTCCTTTATGGATAATA-3′; *antisense*: 5′-GGGGACCACTTTGTACAAGAAAGCTGGGTCCGATAACAAATTGCAGGCATAG-3′, the *attB* sites are underlined). The amplified *attB* fragment was then subcloned into the pDONR221 vector, resulting in the entry clone, and the entry clone was then subcloned again into the expression vector pMDC83, resulting in the pMDC-GmNES-GFP construct under the control of the CaMV35S promoter. A *GmNES* expression cassette was introduced into tobacco (*Nicotiana tabacum cv.* ‘*Samsun*’) by *Agrobacterium*-mediated transformation [17]. Non-transformed tobacco was used as a control. Transgenic lines (T_0_ generation) were selected based on hygromycin resistance, and confirmed by PCR and southern blotting analysis. The positive detected transgenic lines were transferred to plastic pots (diameter = 8 cm, depth = 6.5 cm) in a growth chamber at 25°C under a 16 h light/8 h dark cycle. One month later, plants were transferred from the chamber to a greenhouse, and seeds of the T_1_ generation were obtained. T_1_ seedlings were used for the subsequent insect resistance and antimicrobial assays.

### Subcellular Localization

Tobacco leaf epidermal cells were examined for the green fluorescence of the GmNES::GFP fusion protein with a Leica TCS 4D Confocal Laser Scanning Microscope (CSLM). Green fluorescence corresponding to the GFP fusion protein was detected using a BP515–525 filter after excitation with blue light at 488 nm. Red autofluorescence from chlorophyll was detected using an LP590 filter after excitation with green light at 568 nm.

### Dual-choice Assay and Growth Inhibition Test of Cotton Leafworm

Similar-sized mature full green leaves from transgenic tobacco plants and wild-type plants were collected, cleaned with distilled water, and then placed abaxial side up on moist filter paper in an oblong container (30×50 cm). Twelve third-instar cotton leafworm larvae were released in the middle of the container and, after covering with cling film, the container was incubated at 25°C under long-day conditions (16 h light/8 h dark) with a relative humidity of 70%. The cotton leafworm larvae could easily move toward the leaves inside the container. The number of cotton leafworm larvae on each side of the container was recorded at a range of time points after the start of the experiment. Five separate containers were prepared as replicates. The data were analyzed with SAS 9.2 software (SAS Institute, Cary, NC) [3].

For the growth and development assay, cleaned leaves were placed on moist filter paper in a petri dish (9 cm diameter). Four separate petri dishes were prepared as replicates for each of the four transgenic lines examined. Five third-instar cotton leafworm larvae were released in each petri dish and, after closing, the petri dishes were incubated at 25°C under long-day conditions (16 h light/8 h dark) with a relative humidity of 70%. The leaves were changed regularly on time to ensure normal intake. At each time point, the weight of the cotton leafworm larvae was recorded. The data were also analyzed with SAS 9.2 software (SAS Institute, Cary, NC) [3].
